# Emerging Stent and Balloon Technologies in the Femoropopliteal Arteries

**DOI:** 10.1155/2014/695402

**Published:** 2014-02-04

**Authors:** Georgios Pastromas, Konstantinos Katsanos, Miltiadis Krokidis, Dimitrios Karnabatidis, Stavros Spiliopoulos

**Affiliations:** ^1^Department of Interventional Radiology, Patras University Hospital, 26504 Patras, Greece; ^2^Department of Interventional Radiology, Guy's and St. Thomas' Hospitals, NHS Foundation Trust, London SE1 7EH, UK; ^3^Department of Imaging Sciences Biomedical Engineering, School of Medicine, King's College, London WC2R 2LS, UK; ^4^Department of Radiology, Addenbrooke's Hospital, NHS Foundation Trust, Cambridge CB2 0QQ, UK

## Abstract

Endovascular procedures for the management of the superficial femoral (SFA) and popliteal artery disease are increasingly common. Over the past decade, several stent technologies have been established which may offer new options for improved clinical outcomes. This paper reviews the current evidence for SFA and popliteal artery angioplasty and stenting, with a focus on randomized trials and registries of nitinol self-expanding stents, drug-eluting stents, dug-coated balloons, and covered stent-grafts. We also highlight the limitations of the currently available data and the future routes in peripheral arterial disease (PAD) stent and balloon technology.

## 1. Introduction 

Peripheral arterial disease (PAD) of the lower extremities remains one of the often unrecognized manifestations of systemic arteriosclerosis symptomatically affecting between 3% and 7% of the population and up to one in five patients older than 75 years of age [1]. It has a major harmful impact on quality of life and is an underrecognized marker of multisystem vascular disease. The risk of disease increases two- to threefold for every 10-year increase in age after the age of 40 years, with men developing claudication about twice as commonly as women [2]. Mortality in patients with intermittent claudication (IC) is up to four times that in the nonclaudicants [3]. Approximately 55% of claudicants will die from heart disease, 10% from a stroke, and 10% from abdominal vascular pathology [4, 5]. The strength of association is so strong that even an asymptomatic patient with a slightly reduced ankle brachial index (ABI) of 0.9 has a twofold relative risk of a coronary event [6].

The femoropopliteal artery is the commonest site of involvement in patients with atherosclerotic PAD [7]. Arterial occlusive lesions are typically long with variable degrees of calcification and fibrosis and clinical presentation varies from asymptomatic to lifestyle-limiting intermittent claudication (IC) or critical limb ischemia (CLI). PAD is usually multilevel and femoropopliteal lesions may be combined either with inflow aortoiliac disease usually in middle-aged smokers or with distal infrapopliteal outflow lesions usually in patients presenting with limb-threatening CLI. Percutaneous intervention (angioplasty and/or stenting) is the suggested treatment of choice in the majority of patients with IC or CLI on the basis of its reduced perioperative morbidity and mortality and reduced in-hospital stay, whereas its long-term outcomes are comparable to bypass surgery [8, 9].

## 2. Classification and Management of SFA Occlusive Disease

The TransAtlantic Intersociety Consensus (TASC) has classified four clinically important types of SFA lesions [10, 11].

- TASC type A lesions are a single stenosis ≤10 cm in length or a single occlusion ≤5 cm in length. Endovascular therapy is the treatment of choice for type A lesions.
- TASC type B lesions are classed as multiple lesions (stenoses or occlusions), each ≤5 cm; a single stenosis or occlusion ≤15 cm, not involving the infrageniculate popliteal artery; single or multiple lesions in the absence of continuous tibial vessels to improve inflow for a distal bypass; a heavily calcified occlusion ≤5 cm in length; or a single popliteal stenosis. Endovascular treatment is the preferred treatment for type B lesions.
- TASC type C lesions are multiple stenoses or occlusions totaling >15 cm with or without heavy calcification or recurrent stenoses or occlusions that need treatment after two endovascular interventions. Although surgery is the preferred treatment for good-risk patients with type C lesions, the patient's comorbidities, fully informed patient preference, and the local operator's long-term success rates must be considered when making treatment recommendations for type B and type C lesions.
- TASC type D lesions are classed as chronic total occlusions of the SFA (>20 cm, involving the popliteal artery) or a chronic total occlusion of popliteal artery and proximal trifurcation vessels. Surgery is the treatment of choice for type D lesions.

## 3. History

Despite the high initial technical success rate of femoral percutaneous transluminal angioplasty (PTA), long-term results were rather disappointing. Particularly, in total occlusions and longer segment disease, 6 months patency rates ranged between 30% and 80% [12]. Elastic recoil of the vessel wall, extensive intimal dissection, and restenosis due to intimal hyperplasia remain the major limitations of this technique. Although the additional use of metallic stents has the ability to overcome acute limitations such as elastic recoil and dissection, long-term outcomes may be compromised by the development of neointimal hyperplasia and late restenosis [13]. Several studies reported that excluding critical limb ischemia, long-term outcomes of primary stent implantation and balloon angioplasty were similar [14]. Consequently, for more than a decade, femoropopliteal stent implantation remained a bailout procedure after failed balloon angioplasty. Considering the high rates of recurrence after balloon angioplasty and the inability to offer durable endovascular alternatives, the indication for endovascular treatment of long femoropopliteal segments remained debatable and was definitively not recommended by the first TASC (TransAtlantic Intersociety Consensus) working group [10].

## 4. The Evolution of Stent Engineering: Self-Expanding Stents

The introduction of self-expanding nitinol stents once again improved endovascular treatment of femoropopliteal disease. Old generation balloon-expandable metal stents are no longer used in the femoropopliteal segment as they are susceptible to external compression and longitudinal axis deformation related to restenosis. New generation, self-expanding stents, manufactured from a nickel-titanium alloy (nitinol), demonstrate elastic and thermal memory properties suitable for the infrainguinal arterial bed. Nitinol stents conform better to the unique biomechanical environment of the femoropopliteal artery as a result of their superior resistance to torsion, flexion, extension, contraction, and compression compared to stainless steel [15]. Radial expansion of nitinol stents occurs with in situ intra-arterial stent heating, and, as a 10–20-fold increase in “spring-like” behavior of nitinol is achieved compared with stainless steel alloys, nitinol stents achieve the predefined nominal diameter once deployed without significant foreshortening. To date, nitinol stents are available in lengths up to 25 cm to accommodate long-segment lesions. Stent diameter is oversized at around 1 mm compared to reference vessel size.

Several randomized trials reported the superiority and the improved stent durability of nitinol technology. The Vienna Absolute Trial (balloon angioplasty versus stenting with nitinol stents in the SFA) reported reduced restenosis rates in the stent group compared to balloon angioplasty at 6 months (23.5% versus 43.4%; *P* = 0.05) and 12 months (36.7% and 63.5%; *P* = 0.01) of follow-up [16].

Moreover, primary balloon angioplasty treatment, moderate vessel wall calcifications, and TASC D lesions have been identified as positive predictors of increased provisional femoropopliteal stenting [17]. Overlap of nitinol stents must be minimized or ideally avoided as it relates to increased risk of fracture and site-specific restenosis. Suboptimal nitinol stent expansion is related to heavily calcified eccentric or ring-like concentric plaques. Adequate predilation or even postdilation with a high-pressure balloon is advisable to improve acute angiographic outcomes. Finally, self-expandable stent technology demonstrates specific high resistance to deformation, a property particularly helpful in cases of deployment across vessel segments exposed to high forces of flexion, torsion, and compression such as the adductor's canal and the popliteal artery at the level of the patella.

## 5. Stent-Based Local Drug Delivery and Covered Stent-Grafts

Despite the widespread use of nitinol devices, in-stent restenosis (ISR) due to exaggerated neointimal hyperplasia (NIH) still remains a major drawback of endovascular treatment. Moreover, recurrent restenosis after balloon angioplasty for the treatment of ISR occurs in up to 60% within the first 6 months [18]. The femoral and popliteal arteries are characterized by the highest incidence of postangioplasty vessel restenosis across the various vascular beds of the human body and NIH is detected more often in long-segment occlusions or stenosis and after placement of multiple stents [19]. Following barotrauma, endothelial cells release inflammatory mediators that trigger platelet aggregation, fibrin deposition, and recruitment of leukocytes to the area. These cells express growth factors that promote smooth muscle cells migration from the media to the intima. Smooth muscle cells proliferate within the intima and deposit extracellular matrix, in a process analogous to scar formation. The result is formation of a neointima over the site of injury. An exuberant healing response leads to intimal hyperplasia (thickening) that encroaches on the vessel lumen and causes stenosis. This process is analogous to keloid formation in epithelial wounds and is accelerated by the presence of prosthetic material in the vessel [20].

Since a rapidly increasing number of patients with SFA and popliteal artery disease have been treated by stent implantation in the last few years, we now are confronted with more and more patients presenting with ISR, which is mainly caused by endogenic factors. Since treatment of such restenosis with balloon angioplasty alone has a very high refailure rate, different treatment modalities have been investigated to reduce the high recurrence rate. Modern concepts for effective inhibition of NIH and improvement of long-term patency outcomes include drug-eluting stents (DES) and drug-coated balloons (DCB) that deliver antirestenotic agents to the vessel wall and self-expanding covered stents or stent-grafts that prevent neointimal ingrowth at the site of treatment.

## 6. Drug-Eluting Stents

Drug-eluting stents (DES) are self-expanding nitinol stents that are engineered for sustained drug-elution to the abluminal vessel surface using polymer- or nonpolymer based technologies. Most of current DES platforms achieve prolonged release of sirolimus or an analogue (olimus drug family). Sirolimus (rapamycin) is a natural macrocyclic lipophilic lactone with cytostatic, immunosuppressive, and antibiotic properties which arrests the cell cycle and inhibits smooth muscle cell proliferation [21]. As a result, the concept of combining the advantages of mechanical nitinol scaffolding with the antiproliferative action of drugs seems appealing.

Although several drug-eluting stents have been used in the coronary circulation, only one, the sirolimus eluting SMART stent (Cordis Endovascular, NJ, USA), had the first available data for the periphery. A double-blinded, randomized, controlled trial compared the efficacy of a sirolimus-coated SMART CONTROL stent with that of a similar bare metal SMART CONTROL stent in the femoropopliteal arterial segment. The amount of drug was equivalent to that used in the coronary application for a total of 1.2 mg of sirolimus per stent. The study was performed in two phases, each with a 6-month follow-up, and each with slightly different endpoints. Unfortunately, neither trial achieved a significant reduction in restenosis. Even after 4 years of follow-up, no difference in any metric between the two treatment groups was noted [22, 23]. On the other hand, the Zilver PTX study was the largest, prospective, randomized trial for the endovascular treatment of symptomatic femoropopliteal peripheral artery disease (479 patients) presented to date and the first human study to demonstrate a biologic effect of an antiproliferative agent applied to a stent-based platform, without polymeric coverage, in the femoral artery (Zilver PTX Nitinol Stent, Cook Medical, IN, USA) [24, 25]. The Zilver PTX stent is coated on its outer surface with 3 *μ*g/mm^2^ of paclitaxel and sprayed directly on the metallic surface, without the use of a polymer, binder, or excipient.

Specifically, primary DES deployment demonstrated significantly superior 2-year event-free survival (86.6% versus 77.9%, *P* = 0.02) and primary patency rates (74.8% versus 26.5%, *P* < 0.01) compared with the control group. Additionally, provisional DES demonstrated superior 2-year primary patency compared with the control bare metal stenting group (83.4% versus 64.1%, *P* < 0.01) while achieving superior sustained clinical benefit (83.9% versus 68.4%, *P* = 0.05). Target lesion revascularization-free survival at two years was 86.6% [25]. Nevertheless, a criticism of the trial is that the relatively short lesions included do not represent real-world practice.

## 7. Drug-Coated Balloons

Despite the initial encouraging experience of DES use for femoropopliteal disease, metal implantable devices were associated with problems such as stent fractures, the need for long-term antiplatelet therapy, and stent occlusion. Particularly, when stent occlusion occurs, the endovascular retreating options are very limited [26]. Drug-coated balloons (DCB) emerged as an alternative strategy to avoid restenosis. This stentless approach seems appealing in light of the problems associated with stent fractures and occlusions.

The concept of DCB technology is based on the combination of conventional angioplasty and local drug delivery, as to inhibit neointimal formation. DCB are sophisticated endovascular balloon catheters engineered for acute release of paclitaxel upon immediate contact with the vessel wall using an appropriate excipient, that is, a drug carrier (contrast, urea, and sorbitol) that allows transfer of the lipophobic paclitaxel molecules to the endothelial cells [27]. Paclitaxel is a natural cytotoxic antiproliferative agent (taxanes) with antimitotic properties originally used for cancer chemotherapy. It stabilizes the spindle microtubules during cell mitosis, thereby arresting cell division and leading to cell death [28].

Two German multicenter clinical DCB studies have been published, both using the same coating technology and the same drug, compared with plain balloon angioplasty, and yielded very similar results. The first trial of DCB in the periphery was the THUNDER trial (Local Taxane with Short Exposure for Reduction of Restenosis in Distal Arteries), a double-blinded, multicenter, randomized, controlled trial [29, 30]. The study included 154 patients with femoropopliteal lesions (both stenosis and occlusions) who underwent standard balloon angioplasty, DCB angioplasty, or angioplasty using simple uncoated balloons and paclitaxel dissolved in the contrast medium. Lesion length was 7.3 ± 5.7, 6.5 ± 4.5, and 6.7 ± 5.1 cm for each group, respectively. The angiographic restenosis rates at 6 months were significantly lower among patients treated with the paclitaxel-coated balloons compared with control balloons (17% versus 44%; *P* = 0.01).

In the Femoral Paclitaxel (FemPac) trial [31], 87 patients underwent 1 : 1 randomization between control-uncoated balloon angioplasty and iopromide-paclitaxel-coated balloon angioplasty in the femoropopliteal arteries. Mean lesion length was 5.7 versus 6.1 cm in the two groups, respectively. The coated balloon exhibited significantly lower angiographic restenosis in the treated group than the control group (19 versus 47%; *P* = 0.035) at 6 months.

Hence, both the THUNDER trial and the FemPac trial demonstrated a significant reduction of angiographic binary restenosis and target lesion revascularization at 6 months. Therefore, until today, paclitaxel-coated balloons are recommended for relatively short, noncomplex femoropopliteal lesions, pending the release of more long-term data. However, both trials must be considered within the context of their limitations: small sample sizes, unconventional surrogate end points, heterogeneous patient population and only a short-term angiographic follow-up.

Only recently, the results of the ILLUMENATE First-in-Human study, a prospective, controlled, multicenter study of 50 subjects investigating a new drug-eluting balloon, have been reported in 2013 EuroPCR Scientific Congress. The specific balloon utilizes a rapid-release drug delivery mechanism to infuse paclitaxel (Stellarex drug-coated peripheral angioplasty balloon, Covidien, MA, USA). A total of 58 femoropopliteal lesions (lesion length up to 15 cm) were treated with the Stellarex balloon catheter. Lesions were evaluated angiographically at six months and by duplex ultrasound (peak systolic velocity ratio (PSVR) of ≤2.5) at 6 and 12 months. According to the Kaplan-Meier analysis, the effectiveness endpoint of 12-month primary patency was 87%. Regarding the safety endpoint, the incidence of major adverse events was 4% and 10% at 6 and 12 months, respectively (no deaths, no amputations, only target lesion revascularizations) [32].

Finally, the DANCE (dexamethasone infusion to the adventitia to enhance clinical efficacy after femoropopliteal revascularization) open-label, nonrandomised, single-arm, single-centre pilot study reported the initial results following the application of a new balloon (The Bullfrog drug-delivery catheter, Mercator MedSystems, USA) that deploys a microneedle (130-micron diameter and 0.9 mm length) into the adventitia for the diffusion of anti-inflammatory drug (dexamethasone). The study was presented at this year's Multidisciplinary European Endovascular Therapy (MEET) Congress (9–11 June 2013, Rome, Italy). The Bullfrog balloon is advanced over the wire and subadventitial dexamethasone injections plus contrast are applied every 3 cm, followed by subintimal diffusion and balloon angioplasty or stenting. The study enrolled 20 patients (mean age 66 ± 10 years, 55% diabetics) with femoropopliteal lesions (mean lesion length was 8.9 ± 5.3 cm), suffering mainly from Rutherford class 2 and 3 disease. Results demonstrated an ankle brachial index improvement on discharge, at six months and at 12 months (*n* = 10), and also in Rutherford scores. The authors reported that the 6-month patency rates were comparable to those of drug-eluting stents and drug-eluting balloons for longer lesions. Larger trials investigating the above optimistic novel angioplasty technology are awaited.

## 8. Covered Stent-Grafts 

Covered stents represent an alternative approach to endovascular treatment of SFA lesions. This approach is also known as endovascular bypass, in analogy to a surgical bypass, as the entire region of disease is excluded with a synthetic graft. Stent-grafts consist of a stent platform covered with fabric (Dacron) or polytetrafluoroethylene (PTFE) which act as a barrier against NIH that encroaches the vessel lumen [33]. PTFE-covered stents are engineered with a 30–100 micron pore size to allow for endothelial lining of the stent-graft and vessel healing. Like conventional stents, only self-expanding covered nitinol stents should be used in femoropopliteal interventions. The Viabahn covered stent-graft (Gore & Associates) consists of a nitinol stent frame lined internally with ePTFE. The device has evolved over time, with the addition of a heparin bioactive surface and, more recently, a contoured proximal edge. Results with the Viabahn stent-graft have been studied in comparison to traditional surgical bypass, in relation to nitinol self-expanding stents and in very long femoropopliteal lesions.

An early study of patients with SFA stenosis and symptomatic claudication randomized patients to Viabahn stent grafting or surgical bypass [34]. Patency rates were similar between the two groups after up to 4-year follow-up. Furthermore, the VIPER registry investigated the use of the newer-generation Viabahn stent-grafts, presenting a contoured edge, in long femoropopliteal lesions (mean lesion length: 19 cm). At 1 year, primary and secondary patency rates were 74% and 93% respectively. The main advantage of covered stents is the high immediate technical success rate reported, while overall patency rates were similar for lesions longer than 200 mm, suggesting that Viabahn graft patency may not be affected by lesion length [35]. Finally, according to a multicenter, randomized trial comparing Viabahn stent grafting to uncovered nitinol stent placement in SFA lesions longer than 80 mm, primary patency and TLR events were similar in the 2 arms at 1 year follow-up. Final analysis of the 3-year outcomes is currently underway [36].

More recently, the prospective, randomized, single-blinded, multicenter VIASTAR study was published. The trial enrolled 141 patients with symptomatic PAD to undergo either heparin-bonded, covered stent (VIABAHN, W. L. Gore & Associates, Inc., DE, USA) or, bare metal stent (BMS) treatment. Study outcomes demonstrated that in TASC D lesions the 12-month patency rate was significantly higher for the VIABHAN stent both in the ITT-analysis (VIA 71.3% versus BMS 36.8%; *P* = 0.01) and in the TPP-analysis (VIA 73.3% versus BMS 33.3%; *P* = 0.004). Moreover, freedom from target lesion revascularization was 84.6% for VIABAHN versus 77.0% for BMS (*P* = 0.37), while ankle-brachial index in the VIABAHN group significantly increased at 12 months (0.94 ± 0.23 versus 0.85 ± 0.23; *P* < 0.05). Nonetheless, in the ITT analysis for all lesions, the difference in primary patency rates was not significant [37].

## 9. Biodegradable Stents: An Upcoming Stent Technology

Regardless of any advanced endovascular equipment applied for the management of femoropopliteal atherosclerotic disease, restenosis still consists a major handicap of percutaneous treatment. The idea of a “temporary,” bioabsorbable stent, that will achieve the desirable optimal immediate result while disappears over time discontinuing the stimulus leading to NIH formation, appears encouraging. New bioabsorbable scaffolds are manufactured of resorbable polymers (poly-L-lactic acid) or metal alloys (magnesium) with or without antiproliferative drug elution. Until today, most studies have been performed in patients suffering from coronary disease using drug-coated balloon-expandable stents [38, 39]. The mainstream suggestion is that elution of an antiproliferative drug might be necessary to obtain clinically acceptable results.

Only recently, the results of a multicenter, nonrandomized registry investigating the performance of a biodegradable (REMEDY) stent in SFA lesions have been published [40]. The study enrolled 95 patients with documented symptomatic occlusion or significant stenosis of the SFA. The investigators achieved immediate technical success in 94.6% of cases. All patients were followed up using Doppler's ultrasound and the authors reported that at six-month follow-up, primary patency was 70.9% and TLR was needed in 21.9% of cases.

Despite the initial promising results, data of long-term clinical and angiographic findings in the femoropopliteal arteries are scarce. Multicenter, randomized control trials seem mandatory for further evaluation of this interesting concept. Additionally, the main problem is the unsatisfactory mechanical properties of the biodegradable materials. Current stents are balloon expandable which are not indicated in the SFA environment. Finally, radial force and architecture are another major issue, as nitinol demonstrates a constant outward radial force, whereas polymers do not.

## 10. Conclusion

Endovascular treatment of the femoropopliteal axis remains challenging with respect to immediate, mid- and long-term outcomes. Extensive lesion length makes SFA one of the most difficult vessels to treat. In addition, the SFA is subjected to forces that make interventions more prone to restenosis due to stent deformations and fractures. Plain balloon angioplasty and stenting usually achieve satisfactory intermediate term primary patency rates while relieving symptoms; however, long-term results remain disappointing, especially in diffuse and complex SFA disease. Newer stent designs, stent-grafts, and drug-eluting or bioabsorbable stents may improve upon these results, but restenosis remains a major concern. The indication for endovascular treatment has to be critically discussed with respect to surgical options and conservative medical treatment depending on patient symptoms and comorbidities.
